# M. Edouard Vandezande on Colchicum in Dropsy

**Published:** 1850-02

**Authors:** 


M. Edouard Vandezande *on Colchicum in Dropsy.*—The preparations of *Colchicum Autumnale* are usually employed solely on account of their efficacy in rheumatism and gout. Störck, however, who introduced this plant into the materia medica in 1763, recognised it as a powerful hydragogue, and employed it as a succedaneum to squills. In spite of his recommendations, and of the favourable reports given by Plenck, Quarin, Zacht, Cullen, and others, the employment of Colchicum in dropsy has not extended, and at last has been almost entirely neglected. In later times, the attention of some physicians has been fixed afresh on the power which Störck recognised in colchicum. Among others, Dr. Kennedy, in a memoir presented to the Surgical Society of Ireland, relates that he has, in a large number of dropsical cases, obtained astonishing results from the employment of the vinous tincture of this plant. Having also made use of this preparation in some cases of serous infiltration and accumulation, I have thought it not unprofitable to give a summary account of the results which I have obtained; first, because they are of a nature to lead my professional brethren to have recourse to the hydragogue power of the plant in analogous cases; and also, because the effects of Colchicum have not always been so complete or so manifest as in the cases which I have observed, of which I shall record the principal.

Case i. Jean Bulckaert, a workman, aged 42, of weak constitution, was seized, three years ago, with intermittent fever in Flanders, where, as is well known, this disease is endemic. Having been under treatment for several months in the hospital at Dixmude, he recovered; but, on his returning home, where he could only procure insufficient food, the disease was not long in recurring. Instead of then applying for medical advice, he had recourse to some domestic remedies, which produced no effect. The patient soon presented all the characters of paludal cachexia. The lower extremities, and the whole body in succession, became œdematous; the abdomen was distended, and the respiration impeded. It was when in this condition that Bulckaert first applied to me. After febrifuge tonics and diuretics had been used for six weeks, the œdema disappeared, and the state of the patient's health seemed satisfactory, although there still remained a slight enlargement of the spleen, of which he neglected to get relieved. Last April, two years after the first appearance of the fever, I was again sent for by him. The dropsy had reappeared, but this time it had not been preceded by an attack of fever: there was excessive serous infiltration of all parts of the body, and the urine was slightly albuminous. The same treatment as before was adopted, but without the least success: I then prescribed the wine of Colchicum in daily doses of six grammes (about ʒii), increasing the dose by a gramme daily. The medicine was well borne by the patient: it was increased to 40 grammes daily; this quantity was continued for four days, at the end of which time the patient was free from dropsy. During the latter period of the treatment, the alvine evacuations were abundant, and the quantity of urine was considerably increased.

Case ii. A woman, named Messiaen, became dropsical after an attack of confluent small-pox: the dropsy was preceded, for some days, by paroxysms of intermittent fever. Neither nitric acid nor heat showed the presence of albumen in the urine. I treated her with wine of Colchicum, in doses of eight grammes, without any other medicine. At the end of a fortnight, the anasarca had disappeared, and the patient felt herself completely restored.

Case iii. A poor woman, who had twice been attacked with articular rheumatism, from which had resulted an organic affection of the heart, with habitual dyspnœa, observed her lower extremities to become œdematous, without any known cause. The extreme misery in which this woman was, and her dirty habits, gave but little hope of a prompt result from any treatment whatever. I resolved, however, to give her Colchicum, especially as I thought that the rheumatic diathesis was connected with the production of the dropsy. The medicine was not well borne, in doses of eight grammes. I diminished the dose by onehalf; but, in a few days, I had to discontinue its use entirely, in consequence of the occurrence of vomiting, dry tongue, and intense thirst. Some time after, when the symptoms of gastric irritation had been removed, I again employed the Colchicum in smaller doses, but without any effect. Mild tonics were then prescribed; and, under their use, the œdema at length disappeared. The woman has still œdema of the feet.

Case iv. A miller, addicted to the use of alcoholic liquors, had for some days complained of *courbature*, loss of appetite, and a remarkable diminution of urine, when he perceived symptoms of dropsy. I first saw him on the 15th of last May. The lower extremities, the scrotum, and the hands, were very œdematous; the face was much puffed up; the abdomen was hard and distended; the respiration was impeded. On testing the urine with heat and nitric acid, a large quantity of albumen was detected. The tongue was furred, the stools unfrequent, and the pulse somewhat febrile. The patient complained of no pain in any part of his body. With the object of cleansing the digestive canal, I prescribed an emeto-cathartic, consisting of a decigramme (1½ grain) of tartar emetic, 32 grammes of magnesia, and 250 grammes of water, of which a wineglassful was to be taken every half hour. The patient had several evacuations from the stomach and intestines, without any marked effect on the dropsy. On the 18th of May, wine of Colchicum was ordered in doses of six grammes, and was very easily supported. The next day, I increased the quantity by two grammes, and continued it for ten days. As this treatment produced no amelioration of the symptoms, the patient importuned me to try some other means. The urine being still albuminous, I had recourse to the internal administration of nitric acid, as recommended by Professor Forget. This medicine had the desired effect. In four or five days, there was a remarkable diminution in the size of the scrotum, while the œdema of the extremities decreased; the abdomen became softer, and the face less puffy; the urine was more abundant and less albuminous. In spite of the continued use of the medicine, the patient remained in the same condition for some time; and it was only after using Bestuchef's tincture* for some days, that the œdema and cachexia disappeared, to be succeeded by rapid convalescence, and soon by recovery,

These observations appear to me to be of a nature to induce my professional brethren to have more frequent recourse to the use of wine of Colchicum, in the treatment of certain dropsical affections. I am persuaded, though unable to specify the cases in which its employment is specially indicated, that this medicine may often be advantageously substituted for the hydragogues in most common use. At all events, I would not recommend it in all cases of albuminous nephritis; for there are medicines which act more directly on this disease, and which, therefore, seem to me to be preferable. [*Annales de la Soc. d'Emul. de la Flandre Occidentale*, as quoted in the *Rèv. Méd. Chirurgicale de Paris* for August 1849.]

* Bestuchef's tincture, otherwise called Klaproth's tincture, or ethereal tincture of chloride of iron, is composed of dry perchloride of iron, one part; Hoffmann's liquor, seven parts.

